# A Metabolomics Approach and Chemometric Tools for Differentiation of Barley Cultivars and Biomarker Discovery

**DOI:** 10.3390/metabo11090578

**Published:** 2021-08-26

**Authors:** Claude Y. Hamany Djande, Lizelle A. Piater, Paul A. Steenkamp, Fidele Tugizimana, Ian A. Dubery

**Affiliations:** Research Centre for Plant Metabolomics, Department of Biochemistry, University of Johannesburg, P.O. Box 524, Auckland Park, Johannesburg 2006, South Africa; 201410297@student.uj.ac.za (C.Y.H.D.); lpiater@uj.ac.za (L.A.P.); psteenkamp@uj.ac.za (P.A.S.); ftugizimana@uj.ac.za (F.T.)

**Keywords:** barley, cultivar differentiation, *Hordeum vulgare*, liquid chromatography, mass spectrometry, metabolomics, multivariate data analysis, phenotyping, secondary metabolites, signatory metabolites

## Abstract

One of the ultimate goals of plant breeding is the development of new crop cultivars capable of withstanding increasing environmental stresses, to sustain the constantly growing population and economic demands. Investigating the chemical composition of the above and underground tissues of cultivars is crucial for the understanding of common and specific traits thereof. Using an untargeted metabolomics approach together with appropriate chemometrics tools, the differential metabolite profiles of leaf and root extracts from five cultivars of barley (‘Erica’, ‘Elim’, ‘Hessekwa’, ‘S16’ and ‘Agulhas’) were explored and potential signatory biomarkers were revealed. The study was conducted on seedlings grown for 21 days under identical controlled conditions. An ultra-high performance liquid chromatography coupled to high-resolution mass spectrometry (UHPLC-HRMS) was employed to analyse hydromethanolic leaf and root extracts of barley cultivars. Furthermore, unsupervised and supervised learning algorithms were applied to mine the generated data and to pinpoint cultivar-specific metabolites. Among all the classes of metabolites annotated, phenolic acids and derivatives formed the largest group and also represented the most discriminatory metabolites. In roots, saponarin, an important allelochemical differentially distributed across cultivars, was the only flavonoid annotated. The application of an untargeted metabolomics approach in phenotyping grain crops such as barley was demonstrated, and the metabolites responsible for differentiating between the selected cultivars were revealed. The study provides insights into the chemical architecture of barley, an agro-economically relevant cereal crop; and reiterates the importance of metabolomics tools in plant breeding practices for crop improvement.

## 1. Introduction

Crop production can suffer from diverse environmental pressures, being abiotic or biotic. These pressures are huge setbacks in the goal of meeting the increasing demand for crops in general and barley grain in particular. Aiming to increase crop production, different strategies are often explored. An example is the development of new cultivars, presenting traits or phenotypes of interest, through plant breeding techniques. These cultivars are developed for improved environmental stress resistance, yield and quality traits. Although tremendous advancements have been made in breeding cultivars, one important challenge remains; to develop a cultivar with all desirable phenotypes. Such a task is difficult because of the pleiotropic effect of some genes, the structure of the genetic population, and the linkages that exist between genes on the chromosomes [1]. Moreover, the change of a selected trait may result in the anticipated or unanticipated change of connected traits in either the desired or the opposite direction. Although progress in understanding the systems biology of agronomic traits has been mainly attributed to genetics and genomics, recent plant breeding approaches take into consideration important information provided by the multi-dimensional data related to the epi-genome, genome, transcriptome, proteome, and metabolomes that collectively impact the phenotype.

There is a close relationship between the genotype, the phenotype, and the environment. Phenotypes are influenced by the genotype, the environment, and interactions between the two. Inversely, the interaction between the phenotype and the environment can also affect the genotype [2,3]. Moreover, the variation of the genome affects the phenotype through a series of downstream events on the transcriptome, proteome, and eventually the metabolome. The influence of the genotype on the phenotype facilitates the use of DNA-based markers in plant breeding practices for the selection of important traits. The challenge here is that some genes may be present in the biological system but not fully expressed and therefore, results in ambiguous selection. This can be overcome by the complementary use of metabolite-based markers, given that the plant metabolome constitutes a bridge linking the genotype to the phenotype. In addition, the biochemical actions of metabolites are extensive and include regulation of epigenetic mechanisms and post-translational modifications. Moreover, metabolic information is a closer reflection of the physiological status of the plant as compared to that of the proteome and transcriptome [4,5,6].

The phenotype can be directly modulated by the qualitative and quantitative variation of low-molecular-weight primary- and secondary metabolites, which in that case will be considered as signatory markers for specific trait selection and cultivar identification or characterization [7]. Although plants produce an overwhelming number of secondary metabolites, the application of metabolomics in plant breeding for trait selection or cultivar identification is still undervalued [6]. The use of metabolomic analyses, involving technologically advanced analytical platforms and chemometric tools, offers unique opportunities in elucidating plant biomarkers. Metabolic phenotyping focuses on detecting and quantifying metabolites in a biological system, shedding light on the biochemistry underlying plant metabolism, which also reveals the spatio-temporal distribution of metabolites as well as routes or pathways undertaken (by these metabolites) to perform specific biological functions [8].

As a metabolomics approach, metabolic phenotyping employed alone or in combination with other ‘omics’ technologies, greatly contributes to expand the current knowledge on the important link between the metabolome and the phenotype and hence facilitates the characterization of cultivars. Several studies have demonstrated such applications in cereal crops [6,9,10,11,12,13,14]. One recent study on sorghum cultivars highlighted the use of metabolomics to investigate the underlying biochemistry behind changes in seed colour [13]. Related to the current study, untargeted metabolomics documented the differential metabolite profile of oat cultivars at a seedling stage [14]. Classes of metabolites including amino acids, fatty acids, carboxylic acids, and phenolic compounds were found to be discriminative among the cultivars.

Barley is an important grain crop, ranked fourth among grains in quantity produced. Its importance stems from its use in human food, animal fodder, and as a source of fermentable starch for beer and some distilled beverages. Barley is a widely adaptable crop, grown in temperate and tropical regions as a summer or winter crop respectively. Although not particularly winter hardy, it is also cultivated under cooler conditions. Both total yield by weight and malting quality, probably multigenic traits, are important agronomic properties. Metabolomics can potentially provide a superior analysis of the phenotypic plasticity, conferring different growth and response capacities to different cultivars [6]. In this study, an untargeted metabolomics approach, based on ultra-high performance liquid chromatography coupled to high-resolution mass spectrometry (UHPLC-MS), was employed for chemo-profiling and discrimination between five genetically related (same parents) or unrelated (different parents) cultivars of barley from the Western Cape winter rainfall region of South Africa.

## 2. Results

### 2.1. UHPLC-MS Analyses of Barley Leaf and Root Extracts

**Figure 1:**
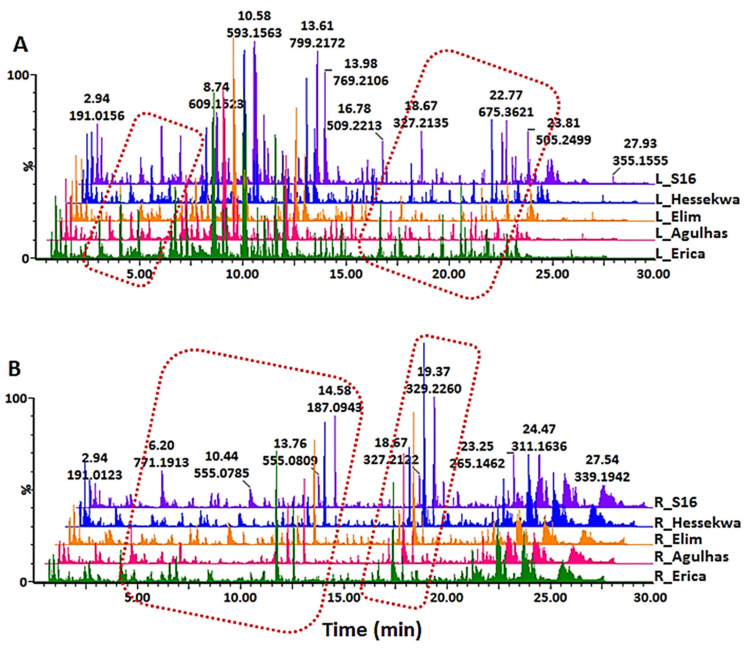
Ultra-high performance liquid chromatography–mass spectrometry (UHPLC-MS) base peak intensity (BPI) chromatograms in negative electrospray ionisation (ESI) mode of (**A**) leaf and (**B**) root extracts from five different barley cultivars (‘Erica’, ‘Elim’, ‘Hessekwa’, ‘S16’ and ‘Agulhas’) under controlled conditions and harvested after 21 days.

Metabolite profiling of five different cultivars of barley was investigated in the study. Leaf and root tissues were harvested after 21 days and the following extraction with cold aqueous methanol, samples were submitted for UHPLC-MS analysis in both positive and negative electrospray ionisation (ESI) modes to provide information on the chemical composition of the extracts. The base peak intensity (BPI) chromatograms from the leaf and root samples are represented in Figure 1A,B respectively, and depict the complexity and diversity of compounds ranging from polar to non-polar in both negative and positive (Supplementary Figure S1A,B) ionisation modes. The visual comparison of chromatographic profiles revealed more distinguishable quantitative as compared to qualitative differences between samples. Examples of such quantitative variations are highlighted with dotted rectangles. Geared toward a more holistic understanding of these cultivar- and tissue-related differences, advanced data processing and chemometric analyses of the complex and multidimensional data were required.

### 2.2. Multivariate Data Analyses: Principal Component- and Hierarchical Clustering Analyses (PCA and HiCA)

**Figure 2:**
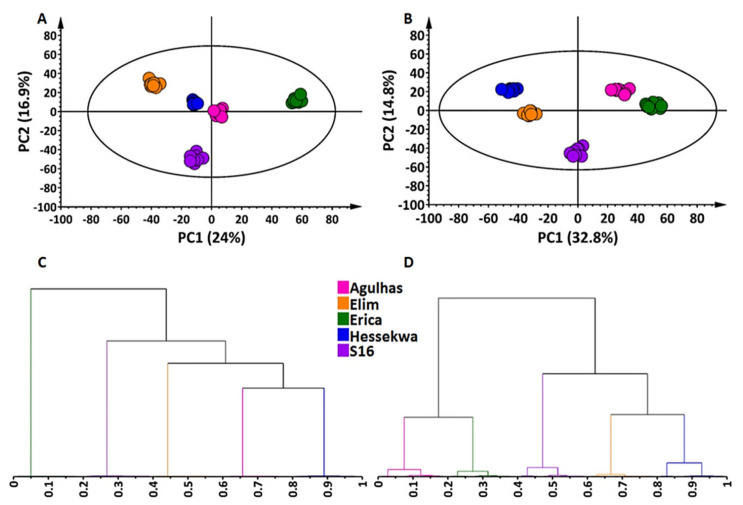
Principal component analysis (PCA) score plots and hierarchical cluster analysis (HiCA) dendrograms of ESI(−) data from leaf and roots extracts of five cultivars of *Hordeum vulgare*. The data was mean-centred and Pareto scaled. The calculated Hoteling’s T2 with a 95% confidence interval is represented by the ellipses present in each PCA scores plot. HiCAs were computed on low-dimensional data derived from the corresponding PCA modelling and highlight sub-clustering formed within the samples. The datasets used to compute these models consisted of 1077 features in leaves and 470 in roots. (**A**) A scores plot (PC1 vs. PC2) of a 4-component (PCA) model explaining 60.3% variation and predicting 51.3% variation in leaves; (**B**) A scores plot (PC1 vs. PC2) of a 5-component model explaining 72.3% variation and predicting 62.4% variation in roots; (**C**) HiCA dendrogram corresponding to the PCA model in (**A**) for leaf extracts, and (**D**) HiCA dendrogram corresponding to the PCA model in (**B**) for root extracts.

A total of 1077 and 1148 features were extracted from leaf data acquired from the UHPLC-MS analyses in negative and positive ionisation modes respectively. For root extracts, the corresponding values were 470 and 781. The extracted data were submitted to multivariate data analyses (MVDA) to further evaluate the metabolite distribution across samples. Two unsupervised chemometric methods, principal component analysis (PCA) and hierarchical clustering analyses (HiCA) were applied to mine the complex and high-dimensional datasets and provided an overview of patterns and trends therein. As seen in Figure 2A (leaves) and Figure 2B (roots), the PCA scores plots showed clear sample groupings with distinct differences between extracts from leaves vs. roots, indicating cultivar-specific variation as well as tissue-specific differences (Figure 2A,B). In the extract from leaves, well-defined clustering was observed in both ESI(−) and ESI(+) data (Figure 2A and Figure S2A). For extracts from roots in ESI(−), two main groups were globally observed: ‘Erica’ and ‘Agulhas’ vs. ‘Elim’-‘S16’-‘Hessekwa’ (Figure 2B). This corresponds with what is known about the genealogy of the cultivars (Section 4.1). A similar observation was not evident in ESI(+) mode, showing no distinct clustering (Figure S2B). Moreover, HiCA displayed the same pattern observed in the corresponding PCA; and more defined sub-clustering was underlined (Figure 2C,D and Supplementary Figure S2C,D).

### 2.3. Unravelling the Cultivar-Specific Metabolic Profile of Barley Leaves and Roots

**Figure 3:**
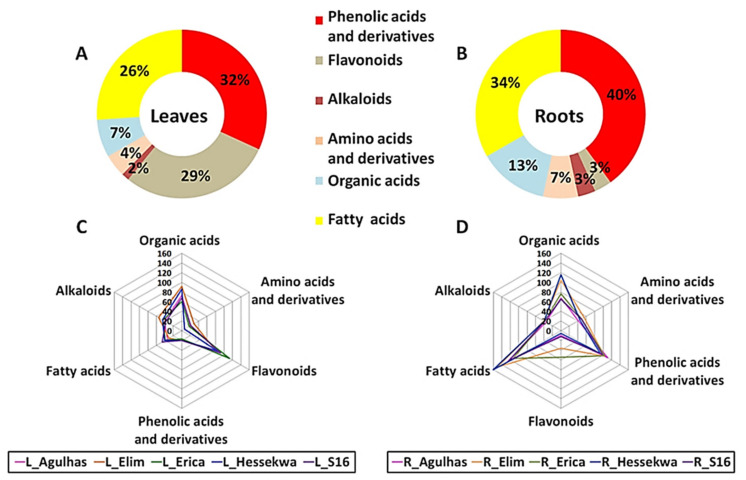
Profiling of metabolites present in methanolic extracts from barley leaf and root extracts. (**A**,**B**) Doughnut charts representing the proportion of all classes of annotated metabolites in leaf—(left) and root extracts (right); (**C**,**D**) Spider plots showing the relative distribution of classes observed in (**A**,**B**) across the cultivars profiled.

Prior to the investigation of differences among the cultivars, global profiling of metabolites was performed to provide a general background of the chemical composition of barley leaf and root extracts irrespective of the cultivars. The UHPLC analyses of leaf and root extracts were performed on combined quality control (QC) samples, which represented the mixture of all cultivars included in the study. The typical workflow involved all the MS features and the aim here was to annotate as many metabolites as possible to have a general overview of the barley metabolome. Compound annotations were based on the accurate masses (*m*/*z*) corresponding to the features obtained after data processing. The empirical formulae generated by the MassLynx^TM^ software (Section 4.4 and Section 4.5) as well as mass fragmentation patterns of these compounds (obtained at different MS collision energies, MS^E^) were compared with those in the databases and literature. Except for hordenine identified with the help of an authentic standard, seventy-four (74) metabolites were annotated as listed in Supplementary Table S1. Among these, 69 were found in the leaves and 30 in the roots; together sharing 24 metabolites. The 75 metabolites were categorised into different classes: phenolic acids and related derivatives, flavonoids, alkaloids, amino acids and derivatives, organic acids and fatty acids (Figure 3).

Annotated metabolites included phenolic acids such as protocatechuic acid hexose, benzylalcohol-hexose-pentose and gallic acids, all annotated in both leaf and root samples as previously described [15,16]. In addition, ferulic-, caffeic- and sinapic acid conjugated with quinic acid or hexose residues were also identified but only in leaf extracts [17,18,19]. In the same class, different isomers of hordatines (benzofurans specific to barley) were identified and annotated in the leaf extracts together with their precursors (*p*-coumaroyl-, feruloyl- and sinapoylagmatines), found in both leaf and root extracts [15,19,20,21,22]. The flavones, luteolin, and apigenin, substituted with mono-, di- and triglycosides as well as cinnamic acid moieties were the principal flavonoids annotated. The prenylated flavanone 6-prenylnaringenin and a flavonoid derivative were also identified [19,20,23]. Saponarin or isovitexin-7-*O*-glucoside was the only flavonoid annotated in extracts from roots. In both leaf and root extracts, the alkaloid hordenine, as well as amino acids such phenylalanine and tryptophan [15,23,24]; organic acids [15,20,25], and fatty acids [15,20,23,26,27,28] were identified (Supplementary Table S1).

#### 2.3.1. Distribution of Metabolite Classes in Leaf and Root Extracts of Barley Cultivars

From the profiling of the metabolites present in leaf and root extracts (resulting in 75 annotated metabolites), phenolic acids and derivatives were the major class of compounds in the extracts from both leaves and roots of barley cultivars; representing 32% and 40% respectively, of the total number of annotated metabolites. In leaf extracts, this was followed by flavonoids representing 29% and then fatty acids making up 26% of all metabolites (Figure 3A). In the roots, phenolic acids and derivatives were followed by fatty acids which represented 34% of the total number of metabolites annotated (Figure 3B). Only one flavonoid, saponarin, an allelochemical, was annotated in the roots. Organic acids, amino acids, and an alkaloid present in both tissue types represented all-together 13% of metabolites in leaves and 23% in roots.

**Figure 4:**
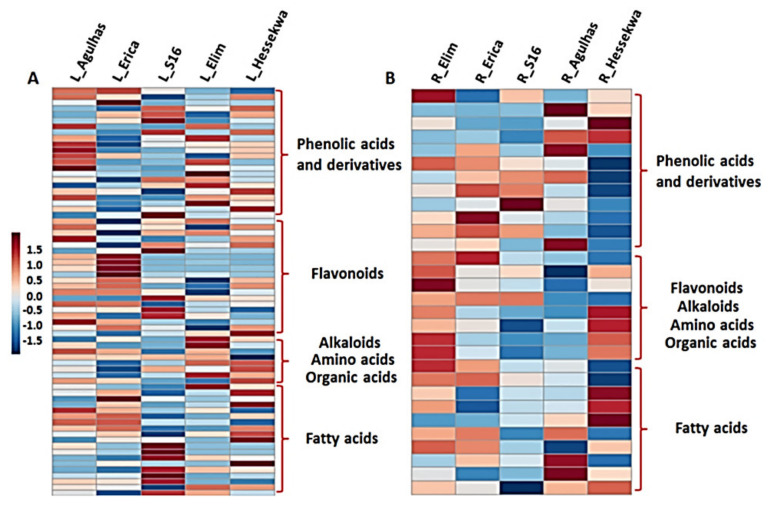
Heatmap overview of the distribution of annotated metabolites present in extracts from (**A**) leaf and (**B**) roots of different barley cultivars: ‘Agulhas’, ‘Erica’, ‘S16’, ‘Elim’ and ‘Hessekwa’. The colour scale was set to default ranging from red (high) to blue (low). L = leaf extracts, R = root extracts. Each cell represents the average peak intensity values (*n* = 9) of specific metabolites within the indicated specific groups.

In addition to differences observed between leaf and root tissues, a differential distribution of these classes of metabolites could also be depicted across cultivars (Figure 3C,D). The distribution was evaluated based on the average relative concentrations (integrated peak areas) of all metabolites constituting each class. In leaf extracts, phenolic acids, organic acids, amino acids, and alkaloids were more prominent in ‘Elim’ as compared to ‘Agulhas’, ‘Erica’, ‘Hessekwa’, and ‘S16’. The highest relative concentration of flavonoids and fatty acids was observed in ‘Erica’ and ‘S16’ respectively (Figure 3C). Similarly, in root extracts, the ‘Elim’ cultivar had the highest level of amino acids and alkaloids, and ‘Erica’ the highest level of flavonoids. Fatty acids and organic acids were relatively more abundant in ‘Hessekwa’ and phenolic acids and derivatives in ‘Agulhas’ (Figure 3D). The diversity observed in the classes of annotated metabolites was also remarkable when investigating individual metabolites across cultivars. This was infographically illustrated in heatmap format (Figure 4), highlighting the differential relative concentrations (average values) of specific metabolites within each metabolite class among cultivars.

#### 2.3.2. Partial Least Squares- and Orthogonal Partial Least Squares-Discriminant Analyses (O)PLS-DA: Differential Metabolite Profiles and Potential Biomarkers

**Figure 5:**
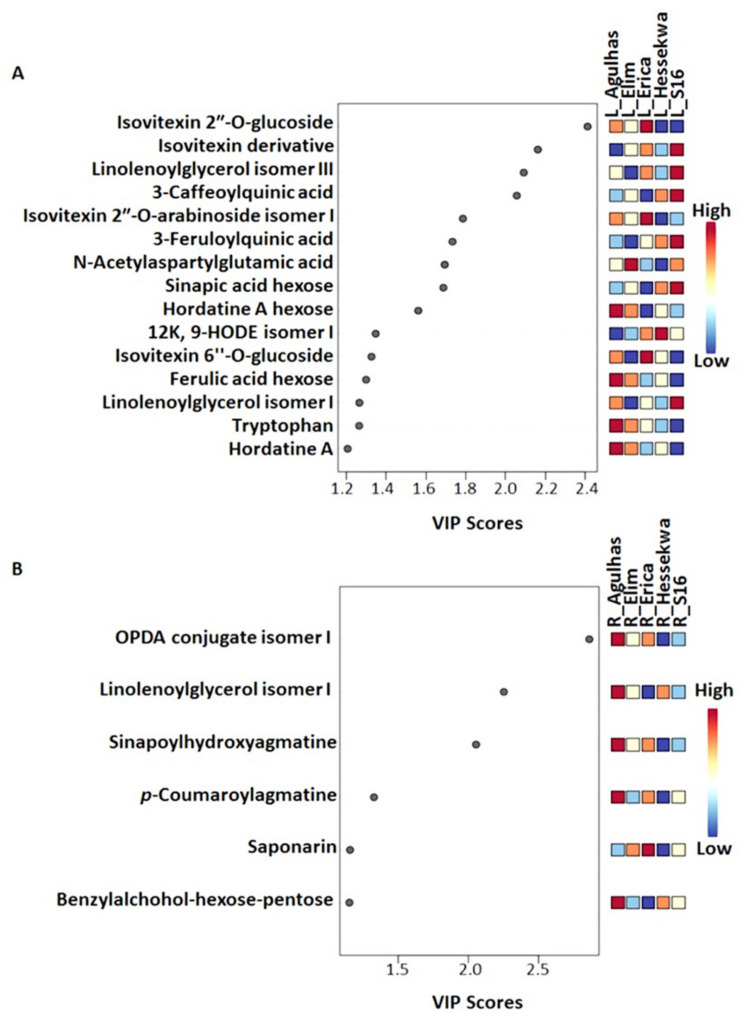
The most important discriminant metabolites (identified by PLS-DA) ranked by variable importance in projection (VIP) scores in component 1. The relative abundance of each important metabolite from leaf (**A**) and root (**B**) samples are indicated with a colour code scaled from blue (low) to red (high). The higher the VIP score, the higher the impact of the metabolite as a discriminant feature among the cultivars. Only the metabolites with a VIP score > 1 were considered.

To select discriminating metabolites between the cultivars, statistical tools, i.e., partial least squares discriminant analysis (PLS-DA) and orthogonal partial least squares-discriminant analysis (OPLS-DA) were performed. PLS-DA is a regression method that can provide descriptive and predictive models, and also discriminates among classes. PLS-DA models for all five cultivars (Supplementary Figure S3) were evaluated using the 10-fold cross-validation which calculated the performance parameters Q^2^ = 92.5%, R^2^ = 99.1% in leaf datasets, and Q^2^ = 91.1%, R^2^ = 97.8% in root datasets. Permutation tests were also applied to validate the generated models. The computed PLS-DA models for leaf and root data were statistically better than the 100 permuted models. Discriminant features identified by PLS-DA were selected based on the variable importance in projection (VIP) scores. The latter estimates the contribution of each variable to the model and features with VIP scores > 1 were considered as important discriminants. Accordingly, a total of 15 and 6 features in leaf and root data respectively, were selected as discriminatory metabolites and are shown in Figure 5, with relative quantitative assessment of each metabolite across the cultivars.

Starting with extracts from leaves, ‘Agulhas’ had the highest level of tryptophan, ferulic acid hexose, hordatine A and its hexosylated conjugate; and the lowest level of 9-hydroxy-12-oxo-10(*E*),15(*Z*)-octadecadienoic acid (12K,9-HODE) isomer I and an isovitexin derivative. In ‘S16’, 3-feruloylquinic acid, 3-caffeoylquinic acid, sinapic acid hexose, the isovitexin derivative, and isomers I and III of linolenoylglycerol were relatively higher compared to the rest of the cultivars. Ferulic acid hexose, hordatine A, isovitexin 6″- and 2″-*O*-glucosides, and tryptophan were the least abundant in the same cultivar. ‘Elim’ and ‘Hessekwa’ had the highest level of *N*-acetylaspartylglutamic acid and 12K,9-HODE isomer II respectively. Except for the isovitexin derivative, higher in ‘S16’, ‘Erica’ had the highest level of all other discriminant flavonoids (isovitexin 2″-*O*-glucoside, isovitexin 2″-*O*-arabinoside isomer I, and isovitexin 6″-*O*-glucoside); and the lowest level of 3-caffeoyquinic acid, sinapic acid hexose, and hordatine A glucose (Figure 5A).

In root extracts, except for saponarin, more predominant in ‘Erica’, ‘Agulhas’ presented the relatively highest level of all discriminant metabolites (Figure 5B). These included: benzylalcohol-hexose-pentose, *p*-coumaroylagmatine, sinapoylhydroxyagmatine, 12-oxo-phytodienoic Acid (OPDA) conjugate isomer I, and linolenoylglycerol isomer I. ‘Hessekwa’ on the other hand had the smallest relative concentrations of OPDA conjugate isomer I, *p*-coumaroylagmatine, sinapoylhydroxyagmatine, and saponarin. The two fatty acids mentioned here displayed the highest VIP scores (>2) in comparison to the rest of the selected metabolites: the higher the VIP score, the greater the contribution of the metabolite to the model classification.

**Figure 6:**
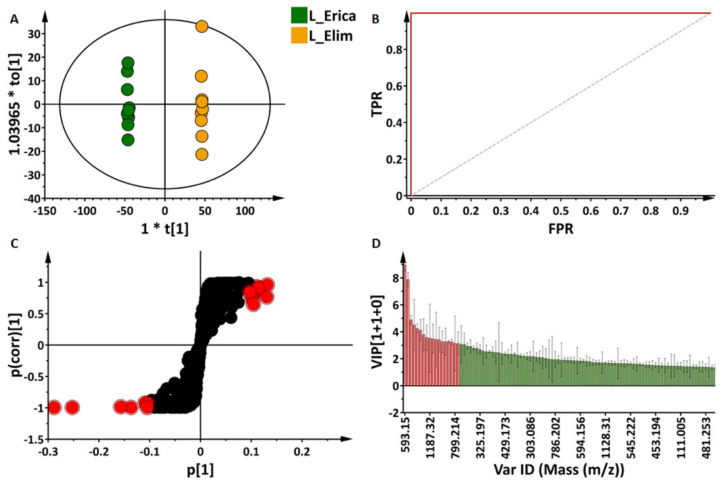
Supervised multivariate data analyses of ultra-high performance liquid chromatography—mass spectrometry (UHPLC-MS) data. Orthogonal partial least squares discriminant analysis (OPLS-DA) modelling and feature selection were performed, based on the unique metabolite profiles of ‘Erica’ and ‘Elim’ leaf extracts in negative ionisation mode. (**A**) OPLS-DA score plots showing a clear separation between the two cultivars. The model is made of 1 predictive component and 1 orthogonal component (R^2^X = 66.4%, R^2^Y = 99.9%, Q^2^ = 99.7%, CV-ANOVA *p*-value = 7.78e^−16^). (**B**) Receiver operator characteristic (ROC) plot summarising the performance of the binary classifier. Perfect discrimination is characterised by a ROC plot showing 100% specificity and sensitivity as above. (**C**) Loading S-plots displaying the features responsible for sample clustering, and are located at the ‘outlier’ ends of the S-plots, highlighted in red here. These features are statistically significant candidates as biomarkers for both cultivars. (**D**) Variable importance in projection (VIP) plot corresponding to the model above and pointing out the mathematical significance of each feature responsible for the discrimination of the cultivars. A VIP score > 1 is considered as significant in the projection and the higher the score values, the more significant the features are.

In addition to PLS-DA, its orthogonal variant, OPLS-DA, a supervised binary classification method, was applied to discriminate between two cultivars at a time and extract potential signatory metabolites corresponding to each cultivar; an example is displayed in Figure 6. The score plot of the generated OPLS-DA model (‘Erica’ vs. ‘Elim’—Figure 6A) displays a clear separation of the two classes of samples under investigation. Different model validation procedures were employed as described in the experimental section. Some of these validation methods include a seven-fold cross-validation (CV) procedure (summarised by the value of quality parameters, cumulative R^2^ and Q^2^ metrics, for explained and predicted variation, respectively). Performance parameters calculated for all the OPLS-DA models generated from leaf and root datasets are similarly presented in Supplementary Table S2.

Furthermore, the receiver operator characteristic (ROC) was applied to evaluate the classification ability of the model (Supplementary Figure S6B). The higher the area under the curve (AUC) the better the model is at distinguishing between the two classes. The high specificity and sensitivity of 100% observed on the curve validate the discriminatory power of the model [29,30].

To select features responsible for the sample discrimination, an evaluation of the OPLS-DA loading S-plots (Figure 6C) was carried out for each pairwise comparison of cultivars. Features with both high correlation and covariation, [p(corr) ≥ 0.5, ≤ −0.5 and (p1) ≥ 0.1, ≤ −0.1] were highlighted. The highlighted variables are significant elements for the biochemical interpretation underlying sample grouping. For statistical purposes, the measurement of the relevance of selected features was further performed with VIP score plots (Figure 6D). Again, only variables with VIP scores higher than the cut-off threshold of 1 were considered as important.

**Figure 7:**
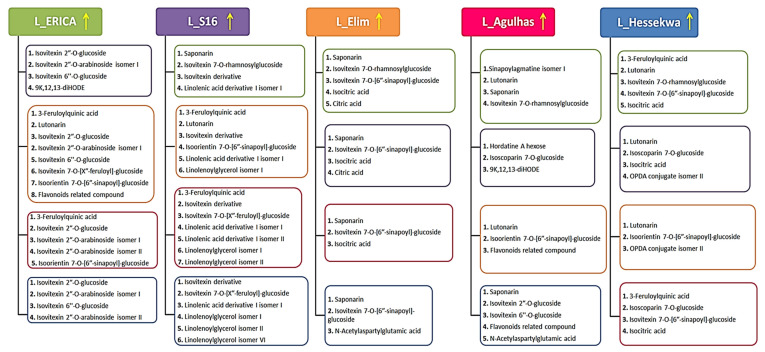
Discriminant metabolites selected from OPLS-DA loadings S-plots generated from the comparison of extracts from leaves from the five cultivars with each other. The arrow indicates the positive correlation of the four groups of metabolites with the corresponding cultivars, e.g., in the first column, comparing ‘Erica’ to ‘S16’, all metabolites in the dark purple rectangle (corresponding to ‘S16’) are positively correlated with ‘Erica’ and negatively correlated with ‘S16’. Using the example in Figure 6 comparing ‘Erica’ and ‘Elim’, eight metabolites were found to be positively correlated to the former, and five were positively correlated to the latter.

In the example provided (Figure 6), ‘Erica’ and ‘Elim’ were compared. From the S-plot (Figure 6C), metabolites positively correlated to each cultivar were selected (as highlighted in red). The VIP scores as well as the corresponding *p*-value and p(corr) of these metabolites are provided in Table S3. The annotation of selected discriminant features was performed as described in Section 2.3 and Section 4.5. These were cross-checked with the metabolites annotated initially which provided additional information on the metabolite composition of barley. Figure 7 (leaf extract data) and Figure S4 (root extract data) summarise and represent all the annotated discriminant metabolites obtained from the S-plots corresponding to each comparison group. Following the same example (‘Erica’ vs. ‘Elim’), 3-feruloylquinic acid, lutonarin, isovitexin 2″-O-glucoside, isovitexin 2″-*O*-arabinoside isomer I, isovitexin 6″-*O*-glucoside, isovitexin 7-*O*-[X″-feruloyl]-glucoside, isoorientin 7-*O*-[6″-sinapoyl]-glucoside and a flavonoid-related compound were positively correlated to ‘Erica’ and negatively correlated to ‘Elim’.

Twenty-six discriminant metabolites were annotated in the leaves and eleven in the roots; together sharing three metabolites; saponarin, citric- and isocitric acids. In the leaves, as part of the flavonoids, lutonarin was positively correlated to ‘Hessekwa’ and negatively correlated to ‘Erica’, ‘S16’, and ‘Elim’. Saponarin, already mentioned as a discriminant metabolite in roots, was also found discriminatory in leaves of all cultivars with the highest intensity present in ‘Elim’. Comparing ‘Erica’ to other cultivars (‘S16’, ’Elim’, ‘Agulhas’ and ‘Hessekwa’) revealed that isovitexin 2″-*O*-glucoside and isovitexin 2″-*O*-arabinoside isomer I were positively correlated to the cultivar while isovitexin 7-*O*-rhamnosylglucoside was negatively correlated. Isoscoparin-7-*O*-glucosides, isovitexin arabinose isomer II, isovitexin-7-*O*-[X″-feruloyl] glucoside, isovitexin-7-*O*-[6″-sinapoyl] glucoside, and the flavonoid-related compound were also found in the leaf extracts to be discriminatory among cultivars. Regarding the fatty acids, the second isomer of OPDA conjugate, 9K,12,13-diHODE, and linolenoylglycerol isomer IV discriminated leaves of ‘Erica’, ‘S16’, ‘Agulhas’ and ‘Hessekwa’. In the roots, *p*-coumaroylhydroxyagmatine and gallic acid monohydrate were selected as discriminant metabolites and positively correlated to ‘Erica’ and ‘Elim’ respectively. 9,12,13-triHODE isomer II was negatively correlated to ‘Erica’ and more prominent in ‘Hessekwa’. In both leaves and roots, citric and isocitric acids were positively correlated to ‘Elim’ and ‘Hessekwa’.

The above-mentioned metabolites, extracted and evaluated using supervised (chemometric) modelling, contribute to cultivar-specific differential metabolite profiles and also to the discrimination between cultivars.

## 3. Discussion

Chromatographically distinct metabolite profiles obtained from barley plants harvested at their third leaf stage of development after 16 days of post-emergence growth are indicative of metabolic variations across the cultivars and tissues. Although limited and not clearly or fully informative, variations observed here throughout UHPLC-MS chromatograms were the first indication of differential chemical compositions between all cultivars. We attempted to annotate as many metabolites as possible to have a general overview of the barley metabolome.

Six main classes of metabolites belonging to different metabolic pathways were annotated in the study. These include phenolic acids and related derivatives, flavonoids, alkaloids, amino acids and derivatives, organic acids, and fatty acids. The biosynthesis of metabolites is dependent on a myriad of factors varying from the plant’s existing genomic and genetic make-up to environmental variation, which contribute to plant phenotypes. Hence, metabolite production is often specific to the type of organism, family, genus, species, cultivars, and the relatedness between those [6,10,31,32,33]. In addition, a differential composition and distribution throughout the plant tissues have also been clearly demonstrated [14,34,35,36,37].

Leaves and roots are two morphologically and functionally different plant tissues. Several studies demonstrated an increase in the production of phenolic acids and flavonoids following high light exposure. In this case, no stress was applied to the plants; however, leaves are naturally more exposed to light as compared to roots [38]. This can explain the presence of a higher number of phenolic compounds in leaves compared to roots. In fact, the main function of leaves is to absorb light and provide energy during photosynthesis. Roots, on the other hand, support the aerial shoot system by anchoring the plant into the ground, and also absorb water and nutrient salts from the soil and conduct them to the upper tissues. These dissimilarities alone can be good contributors to the differential production of any compounds related to their specific functions.

Furthermore, descriptive analyses (PCA and HiCA) allowed us to assess the general structure of the data, hence drew attention to cultivar-related grouping. Sample groupings and cultivar branching observed on the PCA and HiCA respectively, emphasised the existing differential metabolic distribution of barley cultivars. In general, cultivars grouping or branching closely to each other are more related at the metabolome level compared to those farther apart. This was evident in the case of extracts from roots, where two main cluster groups were globally observed: ‘Erica’ and ‘Agulhas’ (sharing the ‘SSG’ parent) vs. ‘Elim’-‘S16’-‘Hessekwa’ that shares ‘Nemesia’ as a parent. However, in the case of the leaf extracts from ‘Agulhas’ and ‘Hessekwa’, these two cultivars seemed to be metabolically more related than the rest, while sample clustering of ‘Agulhas’ and ‘Erica’ (which share a parent) implies differences in the metabolite composition of the extracts. In this context, it is important to note that the metabolome of a biological system can be affected by any variation occurring at transcriptomic—or proteomic levels [6]. As a result, similar genotypes may produce different metabolite profiles. In all cases, differences between the samples originating from the five cultivars were underlined.

From PCA and HiCA to PLS-DA models, clear separation of the five cultivars was observed. This shows the ability to separate and classify multiple cultivars at once, and to identify metabolites responsible for such classification. In addition, from OPLS-DA (where the comparison was reduced to only two cultivars at a time), further metabolites were provided as potential biomarkers. In addition, the chemometric models aided in revealing metabolites (potential markers) that discriminate between cultivars (Figure 7—leaf extracts and Supplementary Figure S4—root extracts). In agriculture and plant breeding practices, such chemometric tools can be applied in quality control assessments and evaluation of soil properties in the field [39,40]; or to detect specific phenotypes [41]. In addition, reports on the application of unsupervised and supervised learning algorithms in metabolomics studies are starting to emerge [10,12,14,42].

Irrespective of the plant tissue, phenolic acids were the major discriminant metabolites, followed by fatty acids and flavonoids. Organic acids as well as amino acids and derivatives were more tissue-dependent as they were the only found discriminants in leaf extracts. Plant metabolites in their diversity can be associated with several agronomical important phenotypic traits, i.e., the quantitative and qualitative occurrence of these particular metabolites is a prediction of specific biological states. These metabolic biomarkers can be characteristic of crop performance traits such as grain or tissue yield, storage properties, morphology, nutritional attributes, sensory qualities, water and nutrient usage, tolerance or resistance to biotic and abiotic stresses, and technological properties traits [43,44,45]. These are important attributes in plant breeding practices.

Phenolic compounds are widely spread throughout the plant kingdom and have been extensively studied in the past years because of their abundance and importance in protecting plants against environmental stresses [46]. Examples of such compounds in the current study may include hordatine A and its precursor *p*-coumaroylagmatine which are more prominent in ‘Agulhas’ leaves and roots respectively, and well known for their strong antifungal properties [47,48]. In barley, the synthesis of phenolic compounds has also been associated with antioxidant activity and allelopathy properties [49,50,51]. The ability of plants to synthesise and release allelochemicals is an important selection characteristic for the reduction of weeds spreading and for the fabrication of bio-pesticides [52,53,54]. Saponarin was regarded as a candidate metabolite marker for the allelopathic characteristic in barley root exudates [51]. Here, the compound was annotated in both leaves and roots, and was found more prominent in the leaves of cultivar ‘Elim’ and the roots of the cultivar ‘Erica’. Flavonoids in general are structurally diverse plant phenolic compounds, playing important roles in plant pigmentation, protection against UV, development, symbiosis, defence, and signalling mechanisms [55,56]. These secondary metabolites were also suggested as strong metabolite biomarkers for the detection of cultivars tolerant to salt stress [57]. In addition to phenolic compounds, alkaloids are also well known as allelopathic metabolites in barley. In fact, the most reported alkaloids in barley, hordenine (a phenethylamine derivative), and gramine (an aminoalkylindole) [58] were the first to be proposed as main contributors to the plant allelopathy [54,59].

C18 unsaturated fatty acids (UFAs) are economically important metabolites, essential for human nutrition. In plants, they play important roles as membrane components, parts of extracellular barriers (e.g., cutin), reservoirs of carbon and energy in triacylglycerol, and modulators in the glycerolipids [60,61]. Moreover, C18 UFAs are also antioxidant precursors and regulators of several bioactive molecules such as jasmonic acid; hence the association to plant defence against biotic and abiotic stresses [61,62]. In wheat, alteration to lipid metabolism was reported as a marker of induced resistance [63].

Organic acids play a central role in the metabolism of plants. They are well known as intermediates in carbon metabolism and are produced through the TCA cycle and glyoxylate metabolism [64,65]. In plants, malic acid is the most accumulated carboxylic acid and plays an additional function as an osmolyte and an anion, balancing the excess of cations in the plant [66]. Organic acids also promote nitrate intake; this was observed in the root tissue of soybean plants in which malic acid was accumulated [64]. As central to various metabolic pathways, organic acids are also precursors for the biosynthesis of many metabolites such as amino acids and lipids [65]. The accumulation of free amino acids is often associated with stress tolerance [67]. For example, amino acid metabolism was reported to regulate the drought stress response in maize [68]. Relatedly, tryptophan was classified as a discriminant metabolite in oat cultivars [14]. In the current study, tryptophan was found discriminating the leaves of all cultivars with the highest concentration in the ‘Agulhas’ and the lowest in ‘S16’. Cultivars rich in amino acids could also be considered during selection programs when interested in their nutritional value for the human diet [69].

Recent advances have confirmed a relationship existing between genetic variants and metabolites that could be useful for metabolic engineering across various plant cultivars. For instance, the biosynthesis of flavonoids in Arabidopsis has been associated with the gene *flavonol 7-O-rhamnosyltransferase*. Increased accumulation of flavonoids in that plant could be correlated with the increase in gene expression levels [70]. As essential components of plant metabolism and phenotypic determinants, the investigation of metabolites is important for the understanding of the biochemistry underlying plant physiological responses. As such, it contributes to the interpretation of interactions and connections which exist between the plant system and the environment. The study showed that metabolomic analyses were effective to observe variation between cultivars that are genetically related as well as non-related cultivars. Uncovering cultivar-related metabolites or a group of functionally related metabolites associated with known agronomical traits and economic attributes would provide additional knowledge that can be useful for breeders in the selection process and development of new cultivars. Although metabolomics provides very insightful information, the combination with other systems biology approaches constitutes a powerful strategy towards a comprehensive understanding of a plant [71,72]. To the best of our knowledge, this study is the first to provide insight into the use of metabolomics and chemometric tools for the identification and differentiation of barley cultivars at an early growth stage. The study provides a global selection of potential biomarkers, and in this specific case, no further assessment of individual metabolites was required. However, for further studies, targeted metabolomics and univariate data analysis can be applied to the multidimensional set of data to investigate the specific changes across cultivars.

## 4. Materials and Methods

### 4.1. Barley Plant Material and Growth Conditions

Barley (*Hordeum vulgare*) seeds were provided by the South African Barley Breeding Institute (SABBI, Bredasdorp, Western Cape, South Africa). Seeds from five different cultivars or lines were from the Western Cape province and were developed as winter rainfall, dryland crops. These included commercial cultivars ‘Erica’ (‘SSG 532’ × ‘Cooper’) and ‘Agulhas’ (‘SSG 532’ × ‘Kinukei 22’) as well as experimental lines ‘S16’ (‘Hessekwa’ × ‘Nemesia’), ‘Elim’ (‘S02/Ferment’ × ‘Nemesia’) and ‘Hessekwa’ (‘S02/Ferment’ × ‘Nemesia’). ‘Erica’ and ‘Agulhas’ thus share one parent (‘SSG 532’), while ‘Elim’ and ‘Hessekwa share both parents (‘SO2/Ferment’ and ‘Nemesia’). ‘S16’ is related to the latter two through the parent ‘Nemesia’. Of the five cultivars, ‘Hessekwa’, ‘Elim’, and S16’ (all sharing ‘Nemesia’ as a parent) are designated as ‘malting’ cultivars.

Plants were grown under optimal, controlled conditions with no applied stressors to minimise biological variation that can be a detrimental factor for metabolomic studies. All cultivars were grown at the same time in a plant growth room under controlled light and temperature conditions: 12 h fluorescent light (≈85 µmol m^−2^ s^−2^) and 12 h dark cycle at 22–27 °C. Prior to cultivation, soil (professional germination mix, Culterra, Muldersdrift, South Africa) was pasteurised at 70 °C and barley seeds were surfaced-sterilised with 70% ethanol for 5 min followed by several rinses with autoclaved distilled water. Following sterilisation, seeds were soaked in autoclaved distilled water for 2 h for imbibition and then planted in wet soil. Plants were watered twice a week; once with distilled water, alternating with a water-soluble chemical fertiliser (Multisol ‘N’, Culterra, Muldersdrift, South Africa). The study was designed to evaluate the metabolite profiles of leaf and root extracts of the different cultivars 21 days after planting (16 days post-emergence). At that time, plants were at their third leaf stage of development (principal stage 1 and secondary stage 3 according to the Zadoks scale, [73]. Three independent biological replicates consisting of at least 3 seedlings of each cultivar were sampled. Upon harvesting, roots and shoots were separated, snap frozen to quench metabolic activity and stored at −80 °C until processed.

### 4.2. Metabolite Extraction and Pre-Analytical Sample Preparation

Each replicate of harvested leaves and roots was extracted with 80% cold aqueous methanol (1:10 *w*/*v* ratio). The mixture was homogenised using an Ultra-Turrax homogeniser and sonicated for 10 s with a probe sonicator (Bandelin Sonopuls, Berlin, Germany) set at 55% power. Homogenates were centrifuged for 20 min at 5100× *g* and 4 °C. Using a rotary evaporator, the hydro-methanolic supernatants were concentrated to 1 mL by vacuum evaporation, transferred to Eppendorf tubes and evaporated at 45 °C to complete dryness in a centrifugal vacuum concentrator (Eppendorf, Hamburg, Germany). Dried extracts were reconstituted with 500 µL of 50% UHPLC-grade methanol (Romil, Cambridge, UK) by vortexing and sonication. For ultra-high performance liquid chromatography-quadrupole time-of-flight mass spectrometry (UHPLC-qTOF-MS) analysis, extracts were filtered through 0.22 µm nylon filters into chromatography vials fitted with 500 µL inserts, capped and kept at −20 °C prior analysis. Each sample was analysed in triplicate.

### 4.3. Sample Analysis Using Ultra-High Performance Liquid Chromatography—High Definition Mass Spectrometry

The UHPLC-qTOF-MS analyses of extracts were performed on a Waters Acquity UHPLC hyphenated with Waters SYNAPT G1 high resolution, accurate mass spectrometer system in V-optics (Waters Corporation, Milford, MA, USA) as a detector. Aqueous-methanol extracts were separated using the Waters HSS T3 C18 column (150 mm × 2.1 mm × 1.8 µm), thermostatted at 60 °C. The T3 column has the advantage of separating compounds ranging from polar to non-polar despite its characteristic as a C18-based reverse-phase column. A binary solvent system made up of water (eluent A) and acetonitrile (Romil Pure Chemistry, Cambridge, UK; eluent B) both containing 0.1% formic acid, was used for the concave gradient elution at a flow rate of 0.4 mL.min^−1^. The elution initiated with 5% B for 1 min and was gradually increased up to 95% B over 24 min. The concentration of B was kept constant at 95% for 2 min and finally, changed back to initial conditions at 27 min. Before the next injection, the conditions were set to allow the analytical column to calibrate for 3 min. The total run time was 30 min and the injection volume for each sample was 2 µL. Each sample was analysed in triplicate. All sample extracts were randomised to reduce the measurement bias. Blanks made of 50% MeOH were included to monitor the background noise and quality control (QC—pooled) samples were used to assess the stability of the LC-MS system. In Figure S5A, the BPI chromatograms of a blank and a QC sample are illustrated in a linked *Y*-axis for the purpose mentioned above. The reliability and the reproducibility of the analysis were confirmed on the PCA models (Figure S5B) where the QC samples clustered closely to each other [74].

The high resolution, accurate mass MS analyses were operated in both negative and positive electrospray ionisation (ESI) modes. The capillary voltage was set at 2.5 kV; the sampling and extraction cone voltages were 40 V and 4.0 V, respectively. The source temperature was fixed at 120 °C and the desolvation temperature at 450 °C. The cone and desolvation gas flows were at 50 L.h^−1^ and 550 L.h^−1^ respectively. Nitrogen was used as the nebulisation gas at a flow rate of 700 L.h^−1^. A mass range of 50 to 1200 *m*/*z* was selected with a scan time of 0.1 s. The reference mass calibrant, leucine enkephalin (50 pg. mL^−1^, [M–H]^−^ = 554.2615 and [M + H]^+^ = 556.2766) was sampled every 15 s and produced an average intensity of 350 counts per scan. The reference allows the processing software (MassLynx XS^TM^, Waters Corporation, Milford, MA, USA) to automatically perform correction of small deviation of centroid masses observed in the sample, from exact mass measurements. This results in a typical mass accuracy of 1 to 3 mDa. In addition, different collision energies (MS^E^, 0–40 eV) were applied to generate fragmentation data to comprehensively extract structural information of detected metabolites. MarkerLynx XS^TM^ software has built-in mathematical functions assisting with noise filtering, peak detection, peak matching, retention time (Rt) alignment and peak integration. These were well described by [74,75].

### 4.4. Data Processing and Data Mining

Visualization and data processing were performed using MassLynx XS 4.1 software (Waters Corporation, Manchester, UK). A visual assessment of mass chromatograms of QC samples vs. blanks showed no ‘contaminant’ peaks from solvents or the system (Figure S5). The centroid ESI positive and negative data were then pre-processed using the MarkerLynx XS application manager tool of MassLynx XS software, creating thus data matrices for further downstream statistical analyses. The application uses the patented ApexPeakTrack algorithm to accurately detect and align peaks [75]. Prior to the calculation of peak intensities, a modified Savitzky–Golay smoothing and integration was applied. Sample normalisation was done based on total ion intensities corresponding to each peak. The processing parameters were set at the retention time (Rt) range of 0.6–25.0 min of the chromatogram, Rt window of 0.2 min, *m*/*z* mass range of 50–1200 Da, mass window of 0.05 Da and mass tolerance of 0.05 Da.

The resulting data matrices (with a noise level below 50%) obtained from processing were exported to SIMCA (soft independent modelling of class analogy) software, version 15 with the ‘Omics’ skin (Sartorius, Stedim Data Analytics AB, Umeå, Sweden) and MetaboAnalyst 5.0 (www.metaboanalyst.ca—online processing of metabolomics data for statistical, functional and integrative analysis, 15 December 2020) for multivariate statistical analyses [76,77]. To maximise the metabolome coverage (and learn the structures in the data), two approaches were carried out; a chemometric approach and a targeted profiling approach. The former is where the compounds are not firstly identified (or annotated), but their spectral patterns are statistically evaluated to extract relevant spectral features that relate to key questions of the study (this was done using SIMCA software). The latter is where most of the metabolites are firstly annotated (or identified) and then various statistical methods are applied to extract information related to the study, changes and/or valuable biomarkers (this was done using the MetaboAnalyst tool). The data was mean-centred and Pareto scaled to balance all features and keep the data structure closed to the original, reduce variable redundancy and correct measurement errors [78]. As a means to explore and extract significant information from the multidimensional large dataset, multivariate data analysis (MVDA) tools were used. These involved unsupervised and supervised learning algorithms well defined by [79].

Principal component analysis (PCA, an unsupervised method) was executed for dimensionality reduction and data exploration; in addition, hierarchical clustering analysis (HiCA) was used as a substructure reduction and allowed to further assess similarities and dissimilarities of trends observed between the five barley cultivars. The cumulative model variation in the matrix X, R^2^X (cum) also known as the ‘goodness of fit’ parameter and the predictive ability, Q^2^ were used to assess models generated in SIMCA software ver. 15 (Sartorius, Stedim Data Analytics AB, Umeå, Sweden). The goodness of fit parameter describes the disparity between the expected values under a statistical model and the observed ones in the datasets. In other words, it describes how well the model fits observations in the dataset. Estimated using cross-validation, the predictive ability describes how accurate the model is in predicting future behaviour [80,81,82]. Models with an R^2^X (cum) and Q^2^ > 50% were considered robust.

As part of supervised learning algorithms, partial least squares-discriminant analysis (PLS-DA) was performed on MetaboAnalyst 5.0 software, and orthogonal projection to partial least squares-discriminant analysis (also known as an orthogonal projection to latent structures-discriminant analysis, OPLS-DA) modelling performed on SIMCA were employed to identify significant/discriminatory ions. PLS-DA provided variable importance in projection scores corresponding to top discriminatory metabolites (VIP score > 1) among the 5 cultivars. With OPLS-DA modelling, comparing two cultivars at a time, the loadings S-plots generated showed the magnitude of each ion’s contribution to the separation |p(1)| in relationship to its significance |p(corr)|. This allowed the selection of metabolites responsible for the differentiation of cultivars when individually compared to each other. Outlier ions at the extremes of the S-plots, the outermost ions with p(corr) ≥ 0.5, ≤ −0.5 and (p1) ≥ 0.1, ≤ −0.1 were investigated as potential biomarkers. Additionally, the analysis of variance testing of cross-validated predictive residuals (CV-ANOVA) was considered to statistically assess the reliability of the computed OPLS-DA models. A good model was indicated with a *p*-value ≤ 0.05. Receiver operator characteristic (ROC) plots were constructed to evaluate the performance of the binary classifiers [30] and VIP plots were generated to assess the statistical significance of the selected discriminant ions [83]. The quality of the MVDA models were determined by diagnostics tools as described in the captions to the figures. Lastly, S-plot-derived potential markers were further investigated using different tests and tools such as the variable importance in projection (VIP) plots, variable trends and descriptive statistics (Table S3).

### 4.5. Metabolite Annotation

The accurate mass as generated by the SYNAPT qTOF MS system was used to derive empirical formulae with the Masslynx XS^TM^ software that were searched against databases for the identification of possible compound matches. These databases included, amongst others, the Dictionary of Natural Products [15], ChemSpider [20], PubChem [23] and the MassBank of North America [26]. Furthermore, MS data were acquired using five different collision energies (MS^E^), varying from 0 to 40 eV to cause fragmentation of the initial ions. This was done to ensure that as much information regarding the structures of the respective compounds could be obtained for downstream structural elucidation and metabolite annotation. MS-based compound annotation, based on accurate mass and mass fragmentation patterns, was as detailed [17], at a metabolite identification level-2 annotation (tentative identification) according to the Metabolomics Standards [84]. Hordenine was identified with the help of an authentic standard (Sigma–Aldrich, Muenchen, Germany). Using average peak intensity values, the coefficient of variation (CV) was calculated for each annotated metabolite to estimate the dispersion around the mean (Table S1).

## 5. Conclusions

The field of plant research has witnessed tremendous advancement over the past years with the development of new technologies capable of generating an impressive amount of interpretable information. Metabolomics is increasingly contributing towards such progress by its application in diverse agricultural domains including plant breeding practices. Aiming to comprehensively uncover and understand the metabolome of a system, metabolomics is indispensable for the understanding of plant metabolism. Using a high-throughput analytical platform (UHPLC-MS), an untargeted metabolomics approach was employed in the study to comprehensively profile methanolic extracts of leaves and roots of barley cultivars grown in areas with colder temperatures and winter rainfall. The annotated metabolites belonged to classes of phenolic compounds, alkaloids, fatty acids, amino acids and derivatives and organic acids. Cultivar- and tissue-specific metabolites occurrence was visible on the mass chromatograms as well as on the generated unsupervised models (PCA and HiCA) used for explorative analysis. The use of supervised, predictive learning algorithms (PLS-DA and OPLS-DA) allowed the successful extraction/selection of metabolic features (or variables) contributing to the differentiation of the cultivars. Annotation of these features provided a list of metabolites that may serve as biomarkers. These metabolite markers may be regarded as links to characteristic performance traits desired in barley breeding for crop improvement and may be incorporated into targeted metabolomics profiling in the case of assessing metabolome phenotypes (metabo-phenotyping) of specific crosses. Considering the complexity of plant metabolism for growth and survival, the investigation of the synergistic and possible antagonistic effects of these metabolites will also contribute to the current knowledge. Lastly, the metabolomic workflow and the identified biomarkers can serve as a foundation for similar studies on diverse barley cultivars grown in different climatic regions.

## Data Availability

The study design information, LC-MS data, data processing and analyses are reported on and incorporated into the main text. Raw data, analyses and data processing information, and the meta-data are being deposited to the EMBL-EBI metabolomics repository—MetaboLights50, with the identifier MTBLS3142 (http://www.ebi.ac.uk/metabolights/MTBLS3142 accessed date 19 July 2021).

## Supplementary Materials

The following are available online at https://www.mdpi.com/article/10.3390/metabo11090578/s1, Figure S1: Ultra-high performance liquid chromatography—mass spectrometry (UHPLC-MS) base peak intensity (BPI) chromatograms (ESI+ mode) of the leaf and root extracts from five different barley cultivars from the Western Cape region of South Africa, Figure S2: Principal component analysis (PCA) score plot models and hierarchical cluster analysis (HiCA) dendrograms of leaf and root extracts of five cultivars of *Hordeum vulgare*, Figure S3: Partial least squares discriminant analyses (PLS-DA) score plots, showing group separation for leaf (**A**) and root (**B**) extracts from barley cultivars ‘Erica’, ‘Agulhas’, ‘S16’, ‘Elim’ and ‘Hessekwa’, Figure S4: Discriminant metabolites selected on OPLS-DA S-plots generated from the comparison of extracts from roots of cultivars with each other, Figure S5: (**A**) Ultra-high performance liquid chromatography—mass spectrometry (UHPLC-MS) base peak intensity (BPI) chromatograms (ESI− mode) of blank and Western Cape quality control (WCQC1) samples. (**B**) A scores plot (PC1 vs. PC2) with QC samples, Table S1: List of all annotated identified metabolites extracted from leaves and roots of the barley cultivars ‘Erica’, ‘Agulhas’, ‘S16’, ‘Elim’ and ‘Hessekwa’, Table S2: Performance parameters calculated for all the OPLS-DA models generated from leaf and root datasets, Table S3: Statistical parameters for the annotated discriminant metabolites selected from OPLS-DA models comparing ‘Erica’ vs. ‘Elim’.
