# Supplementary material for: A Metabolomics Approach and Chemometric Tools for Differentiation of Barley Cultivars and Biomarker Discovery
Source: Metabolites. 2021 Aug 26;11(9):578. doi: 10.3390/metabo11090578 (PMC8466441; doi:10.3390/metabo11090578)
Supplement: Supplementary file 1 [file metabolites-11-00578-s001.zip › metabolites-1288992-supplementary.pdf]

Supplementary File

A Metabolomics Approach and Chemometric Tools for Differentiation of Barley Cultivars and Biomarker Discovery

Claude Y. Hamany Djande, Lizelle A. Piater, Paul A. Steenkamp, Fidele Tugizimana and Ian A. Dubery*.

Research Centre for Plant Metabolomics, Department of Biochemistry, University of Johannesburg, P.O. Box 524, Auckland Park, Johannesburg 2006, South Africa; 201410297@student.uj.ac.za (C.Y.H-D.); ftugizimana@uj.ac.za (F.T.); psteenkamp@uj.ac.za (P.A.S.); lpiater@uj.ac.za (L.A.P.)
* Correspondence: idubery@uj.ac.za; Tel.: +27-11-5592401

Figure S1. Ultra-high performance liquid chromatography - mass spectrometry (UHPLC-MS) base peak intensity (BPI) chromatograms (ESI+ mode) of leaf - and root extracts from five different barley cultivars from the Western Cape region of South Africa;

Figure S2. Principal component analysis (PCA) score plot models and hierarchical cluster analysis (HiCA) dendrograms of leaf and roots extracts of five cultivars of *Hordeum vulgare.*

Figure S3. Partial least squares discriminant analyses (PLS-DA) score plots, showing group separation for leaf (**A**) and root (**B**) extracts from barley cultivars 'Erica', 'Agulhas','S16','Elim'and 'Hessekwa'.

Figure S4. Discriminant metabolites selected on OPLS-DA S-plots generated from the comparison of root extracts from five cultivars with each other.

Figure S5: (A) Ultra-high performance liquid chromatography - mass spectrometry (UHPLC-MS) base peak intensity (BPI) chromatograms (ESI- mode) of blank and Western Cape quality control (WCQC1) samples. **(B)** A scores plot (PC1 *vs.* PC2) with QC samples.

Table S1. List of all annotated / putatively identified metabolites extracted from leaves and roots of the barley cultivars 'Erica', 'Agulhas','S16','Elim'and 'Hessekwa'.

Table S2. Performance parameters calculated for all the OPLS-DA models generated from leaf and root datasets.

Table S3. Statistical parameters for the annotated discriminant metabolites selected from OPLS-DA models comparing 'Erica' *vs*. 'Elim'.

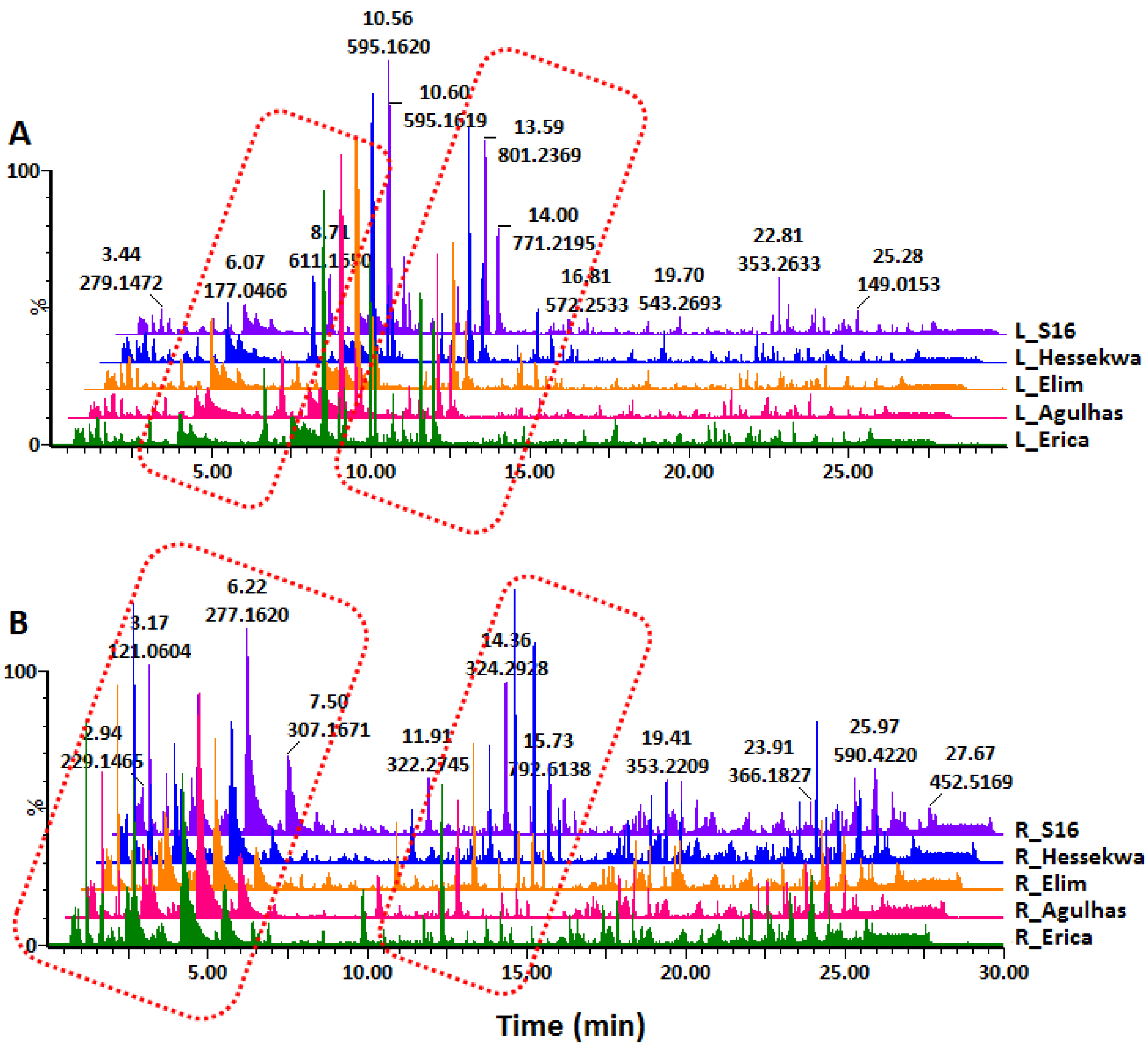

Figure S1. Ultra-high performance liquid chromatography - mass spectrometry (UHPLC-MS) base peak intensity (BPI) chromatograms (ESI+ mode) of (**A**) leaf and (**B**) root extracts from five different barley cultivars from the Western Cape region of South Africa cultivated under controlled conditions and harvested after 21 days.

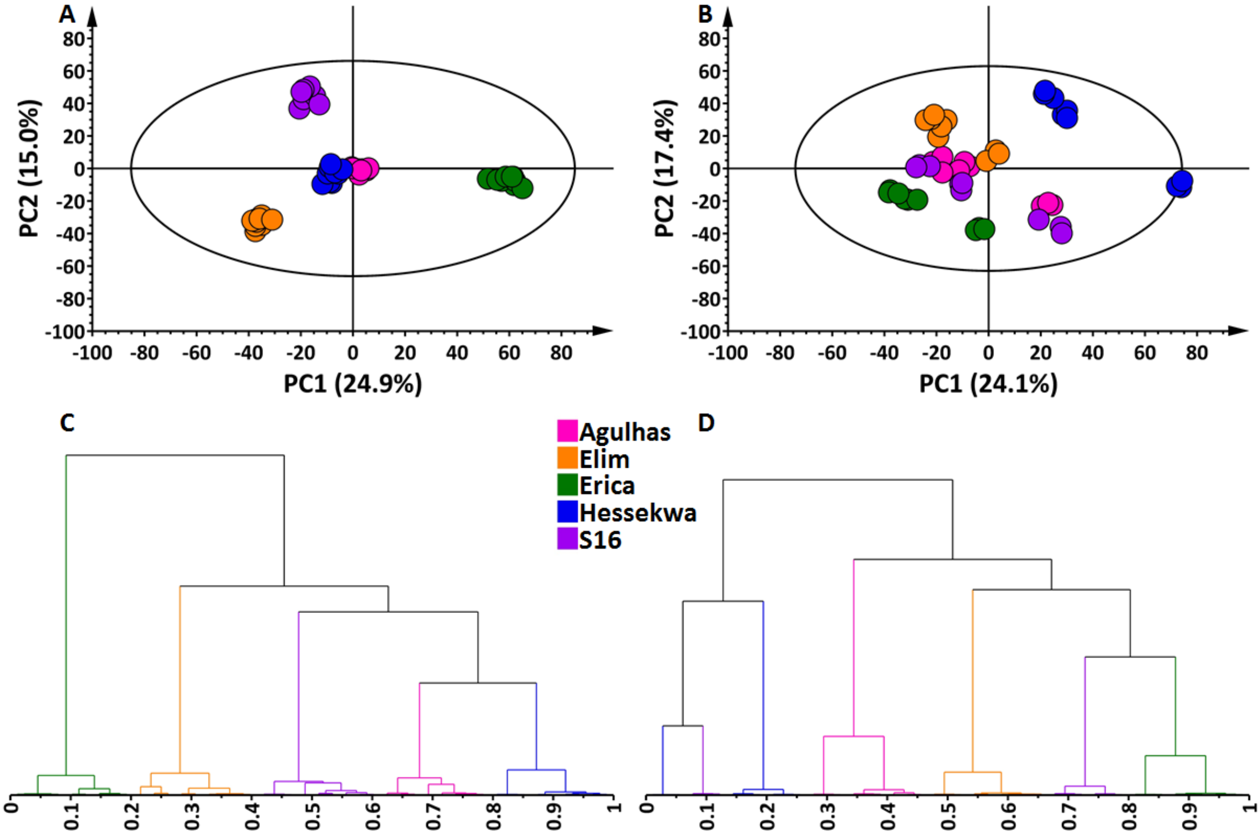

Figure S2. Principal component analysis (PCA) score plots and hierarchical cluster analysis (HiCA) dendrograms of ESI(+) data from leaf and roots extracts of five cultivars of *Hordeum vulgare*. The calculated Hoteling's T2 with a 95% confidence interval is represented by the ellipses present in each PCA scores plot. HiCAs are computed on low-dimensional data derived from the corresponding PCA modeling and highlight sub-clustering formed within the samples. The datasets used to compute these models consisted of 1148 features in leaves and 781 in roots. (**A**) A scores plot (PC1 *vs* PC2) of a 6-component (PCA) model explaining 66.5% variation in a *Pareto*-scaled, and predicting 51.3% variation in leaves; (**B**) A scores plot (PC1 *vs* PC2) of a 5-component model explaining 72.0% variation in a *Pareto*-scaled, and predicting 63.3% variation in roots; (**C**) HiCA dendrogram corresponding to the PCA model in **A,** and (**D**) HiCA dendrogram corresponding to the PCA model in **B** for root extract.

Table S1. List of all annotated metabolites extracted from leaves and roots of the barley cultivars 'Erica', 'Agulhas','S16','Elim'and 'Hessekwa'.

No	Annotated Metabolites	Rt (min)	*m/z* (ESI-)	*m/z* (ESI+)	Leaf CV (%)	Root CV (%)	References
	**Hydroxybenzoic acids**						
1	Protocatechuic acid hexose	1.69	315.0749		1.22	17.73	[16]
2	Benzylalcohol-hexose-pentose	5.66	401.1405		8.15	10.95	[15,16]
3	Gallic acid monohydrate	12.56	187.0942		20.50	2.954	[16]
	**Hydroxycinnamic acid and derivatives**						
4	Ferulic acid hexose	1.76	355.0676		16.03		[18,19]
5	3-Caffeoylquinic acid	2.16	353.0865		10.37		[17-19]
6	3-Feruloylquinic acid	4.08	367.1053		10.11		[17-19]
7	Sinapic acid hexose	5.37	385.1129		9.72		[65,66]
8	4-Feruloyquinic acid	7.44	367.0996		10.11		[18,19]
	**Hydroxycinnamic acid amides**						
9	*p*-Coumaroylputrescine	2.46		235.1346		4.29	[15,20]
10	*p*-Coumaroylhydroxyagmatine	2.66	291.1426	293.1544	9.37	10.05	[19,21,22]
11	Feruloylagmatine isomer I	3.29		307.1745		5.15	[19,21,22]
12	Feruloylhydroxyagmatine	3.54		323.1679	7.65	11.68	[19,21,22]
13	*p*-Coumaroylagmatine	4.23		277.1581	5.28	12.86	[19,21,22]
14	Feruloylagmatine isomer II	5.49		307.1705	9.24	17.40	[19,21,22]
15	Sinapoylagmatine isomer I	6.41		337.1794		8.27	[19,21,22]
16	Sinapoylhydroxyagmatine	6.54	351.1222		18.61	11.14	[19,21,22]
17	Sinapoylagmatine isomer II	7.89		337.1819		9.20	[19,21,22]
	**Benzofurans**						
18	Hordatine B isomer I	7.60	579.2993	581.3153	8.57		[19,21,22]
19	Hordatine B isomer II	7.72		581.3143	10.72		[19,21,22]
20	Hordatine D	7.82		641.1716	5.72		[19,21,22]
21	Hordatine A	7.93	549.2915	551.3013	8.20		[19,21,22]
22	Hordatine C	8.25	609.3073	611.3342	20.28		[19,21,22]
23	Hordatine C hexose isomer I	3.56	771.2131		12.40		[19,21,22]
24	Hordatine B hexose	4.03	787.3706	743.382	14.08		[19,21,22]
25	Hordatine A hexose	4.34	757.3595	713.3724	3.10		[19,21,22]
26	Hordatine C hexose isomer II	5.41	771.1981		12.67		[19,21,22]
	**Flavonoids**						
27	Isoorientin 7-O-glucoside / Lutonarin	6.74	609.1421	611.1583	16.91		[19,20,23]
28	Isovitexin -7-O-glucoside / Saponarin	8.64	593.1534		7.23	14.58	[15,19,20]
29	Isovitexin 7-O-rhamnosylglucoside	9.04	739.23		13.70		[15,19,20]
30	Isoscoparin 7-O-glucoside	9.20	623.1533		9.74		[19]
31	Isovitexin 7-O-[6″-sinapoyl]-glucoside 4′-O-glucoside	9.40	961.2755		6.11		[19]
32	Isovitexin derivative	9.91	611.2522		8.23		[19]
33	Isovitexin 2″-O-glucoside	10.01	593.1427	595.1627	11.76		[19]
34	Isovitexin 2″-O-arabinoside isomer I	10.15	563.134	565.1579	5.01		[19]
35	Isovitexin 6''-O-glucoside	10.21		595.1653	6.92		[19]
36	Isovitexin 2″-O-arabinoside isomer II	10.43	563.1417		2.53		[19]
37	Luteolin 7-O-arabinosylglucoside	10.91	579.1371		27.07		[19]

38	Isovitexin 7-O-[6″-sinapoyl]-glucoside	11.59	799.2158	801.2339	35.63		[19]
39	Isoscoparin 7-O-[6″-sinapoyl]-glucoside	11.69	829.2297		8.04		[19]
40	Isovitexin 7-O-[X″-feruloyl]-glucoside	11.98	769.2041	771.2312	10.22		[19]
41	Apigenin 7-O-arabinosylglucoside	12.06	563.14		8.92		[19]
42	Isoscoparin 7-O-[6″-feruloyl]-glucoside	12.10	799.2159		3.96		[19]
43	Chrysoeriol 7-O-arabinosylglucoside	12.37	593.144	595.163	3.21		[19]
44	6-Prenylnaringenin	19.18	339.2123		8.51		[19]
45	Isoorientin 7-O-[6″-sinapoyl]-glucoside	10.71	815.2056		15.62		[19]
46	Flavonoid-related compound	11.03		787.2163	7.02		[19]
	**Alkaloids**						
47	Hordenine	1.17		166.1139	3.50	7.65	Authentic Standard
	**Amino acids and derivatives**						
48	Phenylalanine	1.68	164.0699	166.0823	11.72	9.08	[16,20,24]
49	Tryptophan	2.51	203.0767	205.0928	10.64	5.77	[16,24]
50	N-Acetylaspartylglutamic acid	6.03	303.0826		6.19		[15-23]
	**Organic acids compounds**						
51	Isocitric acid	0.92	191.0038		9.62	9.10	[25]
52	Malic acid	1.02	133.012		6.69	9.56	[25]
53	Citric acid	1.16	191.0066		10.53	9.11	[25]
54	Succinic acid	1.20	117.0103		8.21	10.89	[15, 25,29]
55	Citric acid derivative	1.41	306.1123		14.58		
	**Fatty acids and derivatives**						
56	α-Linolenoyl ethanolamide / N-(9Z,12Z,15Z-octadecatrienoyl)-ethanolamine	9.87		322.2772		4.21	[15]
57	Linoleoyl ethanolamide / N-(2-Hydroxyethyl)linoleamide /N-(2-Hydroxyethyl)-9,12-octadecadienamide	12.33		324.2901		16.37	[15]
58	(10E,15Z) 9,12,13-trihydroxyoctadeca-10,15-dienoic acid isomer I (9,12,13-TriHODE)	16.57	327.2131		14.32		[20,26]
59	9,12,13-TriHODE isomer II	16.67	327.2170		15.66	18.79	[20,26]
60	9,12,13-TriHODE isomer III	16.79	327.2132		15.66		[20,26]
61	Trihydroxyoctadecenoic acid	17.38	329.2278		12.41	6.39	[20,26]
62	9-Oxo-12,13-dihydroxy-10E,15Z-octadecadienoic acid (9K,12,13-diHODE)	17.61	325.1967		4.57	9.22	[20,26]
63	OPDA conjugate isomer I	19.61	309.2024		21.80	10.61	[27]
64	OPDA conjugate isomer II	19.68	309.1991		8.67	16.96	[27]
65	9-Hydroxy-12-oxo-10(E),15(Z)-octadecadienoic acid isomer I (12K, 9-HODE)	20.09	309.2034		11.66		[27]
66	12K, 9-HODE isomer II	20.59	309.2019		11.73	6.56	[27]
67	Linolenic acid derivative I isomer I	20.77	675.3553		12.13		[23]
68	12-Oxo-Phytodienoic Acid (12-OPDA)	21.11	291.1946		12.42		[27,28]
69	Hydroxyoctadecadienoic acid / Hydroxylinoleic acid	22.37	295.2256		11.27	5.05	[28]
70	Linolenic acid derivative I isomer II	21.07	675.3615		6.13		[23]
71	Linolenoylglycerol / monolinolenin Isomer I	20.81		353.2632	7.90	17.91	[15,20,23]
72	Linolenoylglycerol / monolinolenin Isomer II	21.11		353.2595	10.06		[15,20,23]

73	Linolenoylglycerol / monolinolenin Isomer III	21.27		353.2644	11.42	[15,20,23]
74	Linolenoylglycerol / monolinolenin Isomer VI	21.94		353.2625	10.26	[15,20,23]
75	Linolenic acid derivative II	22.69	445.2328		7.25	[23]

***Rt:** Retention time; ***m/z*:** mass-to-charge ratio; **ESI-/+:** electrospray ionisation modes; **PLS-DA:** partial least square-discriminant analysis; **OPLS-DA:** orthogonal projection to latent structures-discriminant analysis**.**

CV: coefficient of variation.

**Metabolites were annotated corresponding to the Metabolomics Standards Initiative (MSI) level 2, based on accurate mass values, empirical formulae and mass fragmentation data (section. *4.3*).

*** The annotation of metabolites was conducted on the combined quality control samples (QCs) for leaf and root extracts. For phenotyping, two supervised learning algorithms were employed to select discriminant metabolites; the PLS-DA and OPLS-DA as mentioned under ‘Materials and Methods’.

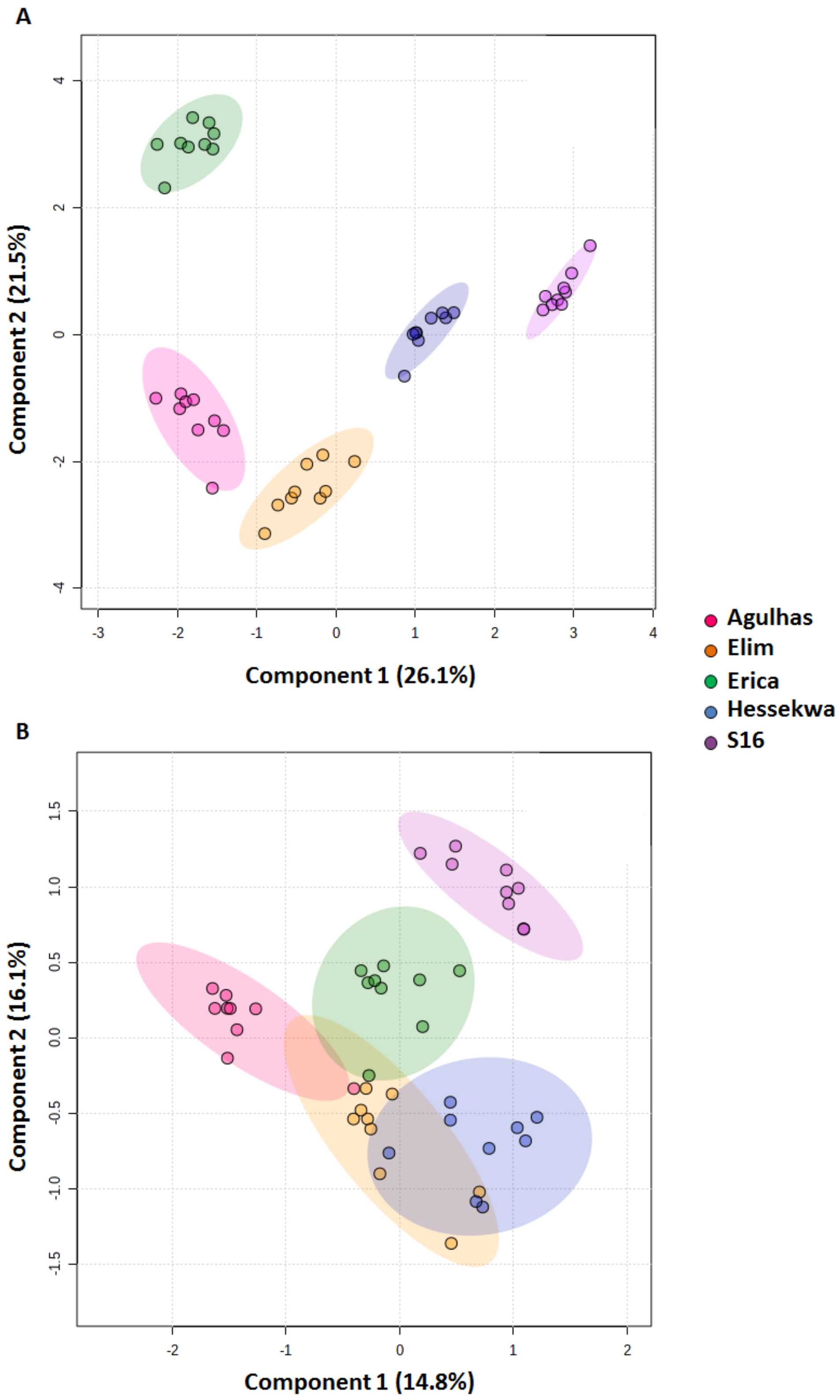

Figure S3. Partial least squares discriminant analyses (PLS-DA) score plots, showing group separation for leaf (**A**) and root (**B**) extracts from barley cultivars 'Erica', 'Agulhas','S16','Elim'and 'Hessekwa'. (Non-redundant data from both positive and negative ionisation modes combined was used to construct the plots).

Table S2. Performance parameters calculated for all the OPLS-DA models generated from leaf and root datasets.

Leaf extracts								
	ESI Negative				ESI Positive			
**Cultivars**	**R^2X**	**Q^2**	**CV-ANOVA**	**Cultivars**	**R^2X**	**Q^2**	**CV-ANOVA**	**Cultivars**
**Erica**	0.628	0.997	7.78e^{-16}	**Elim**	0.591	0.995	5.75e^{-15}	**Erica**
**Erica**	0.538	0.995	1.38e^{-14}	**Hessekwa**	0.529	0.992	1.48e^{-15}	**Erica**
**Erica**	0.498	0.992	1.48e^{-13}	**Agulhas**	0.652	0.979	9.11e^{-11}	**Erica**
**Erica**	0.581	0.994	2.30e^{-14}	**S16**	0.575	0.991	3.63e^{-13}	**Erica**
**Elim**	0.472	0.987	3.87e^{-12}	**Hessekwa**	0.471	0.986	7.88e^{-12}	**Elim**
**Elim**	0.508	0.993	7.97e^{-14}	**Agulhas**	0.695	0.986	1.53e^{-09}	**Elim**
**Elim**	0.542	0.993	1.10e^{-13}	**S16**	0.52	0.988	2.04e^{-12}	**Elim**
**Hessekwa**	0.459	0.983	8.31e^{-12}	**Agulhas**	0.639	0.971	7.24e^{-10}	**Hessekwa**
**Hessekwa**	0.493	0.99	7.31e^{-13}	**S16**	0.476	0.984	1.69e^{-11}	**Hessekwa**
**Agulhas**	0.474	0.989	1.81e^{-12}	**S16**	0.656	0.98	6.15e^{-11}	**Agulhas**
**Root extracts**								
	ESI Negative				Positive			
**Cultivars**	**R^2X**	**Q^2**	**CV-ANOVA**	**Cultivars**	**R^2X**	**Q^2**	**CV-ANOVA**	**Cultivars**
**Erica**	0.664	0.997	5.93e^{-16}	**Elim**	0.591	0.986	7.71e^{-12}	**Erica**
**Erica**	0.621	0.995	4.86e^{-18}	**Hessekwa**	0.721	0.993	9.21e^{-14}	**Erica**
**Erica**	0.462	0.986	5.86e^{-12}	**Agulhas**	0.595	0.984	1.44e^{-11}	**Erica**
**Erica**	0.608	0.992	2.15e^{-13}	**S16**	0.625	0.984	1.53e^{-11}	**Erica**
**Elim**	0.512	0.989	1.42e^{-12}	**Hessekwa**	0.66	0.989	1.60e^{-12}	**Elim**
**Elim**	0.612	0.993	8.76e^{-14}	**Agulhas**	0.623	0.993	1.03e^{-13}	**Elim**
**Elim**	0.543	0.991	3.24e-13	**S16**	0.616	0.969	1.10e-09	**Elim**
**Hessekwa**	0.638	0.999	3.08e-14	**Agulhas**	0.636	0.987	3.77e-12	**Hessekwa**
**Hessekwa**	0.597	0.994	1.76e-14	**S16**	0.697	0.981	5.65e-11	**Hessekwa**
**Agulhas**	0.578	0.992	2.19e-13	**S16**	0.504	0.979	8.18e-11	**Agulhas**

Table S3. Statistical parameters for the annotated discriminant metabolites selected from OPLS-DA models comparing leaf extracts from ‘Erica’ *vs*. ‘Elim’.

Discriminant metabolites	VIP scores	p-score	p(corr)	L_Erica	L_Elim
Isovitexin 2″-O-glucoside	8.97	3.19e^{-17}	-0.99453	+	-
Isovitexin 2″-O-arabinoside isomer I	7.87	1.53e^{-17}	-0.99546	+	-
Saponarin	4.51	1.82e^{-4}	0.768655	-	+
Lutonarin	3.92	8.76e^{-09}	-0.94024	+	-
Isocitric acid	3.79	1.08e^{-07}	0.913522	-	+
Isovitexin 7-O-rhamnosylglucoside	3.56	2.36^{-07}	0.906435	-	+
Citric acid	3.43	3.81e^{-05}	0.815452	-	+
3-Feruloylquinic acid	3.40	3.23e^{-08}	-0.92682	+	-
Isoorientin 7-O-[6″-sinapoyl]-glucoside	3.26	2.31e^{-08}	-0.97072	+	-
Isovitexin 6''-O-glucoside	3.25	1.08e^{-17}	-0.99531	+	-
Flavonoid-related compound	3.29	3.52e^{-12}	-0.90728	+	-
Isovitexin 7-O-[6″-sinapoyl]-glucoside	3.14	3.64e^{-4}	0.746233	-	+

Isovitexin 7-O-[X″-feruloyl]-glucoside	3.13	$1.52e^{-05}$	-0.83667	+	-

(+) Positively correlated to cultivar. (-) Negatively correlated to the cultivar. The VIP score threshold of > 1 was selected based on a p(corr) ≥ 0.5, ≤ -0.5. CV-Anova *p*-scores (derived from SIMCA software) indicate the significance of specific metabolite levels to the OPLS-DA model.

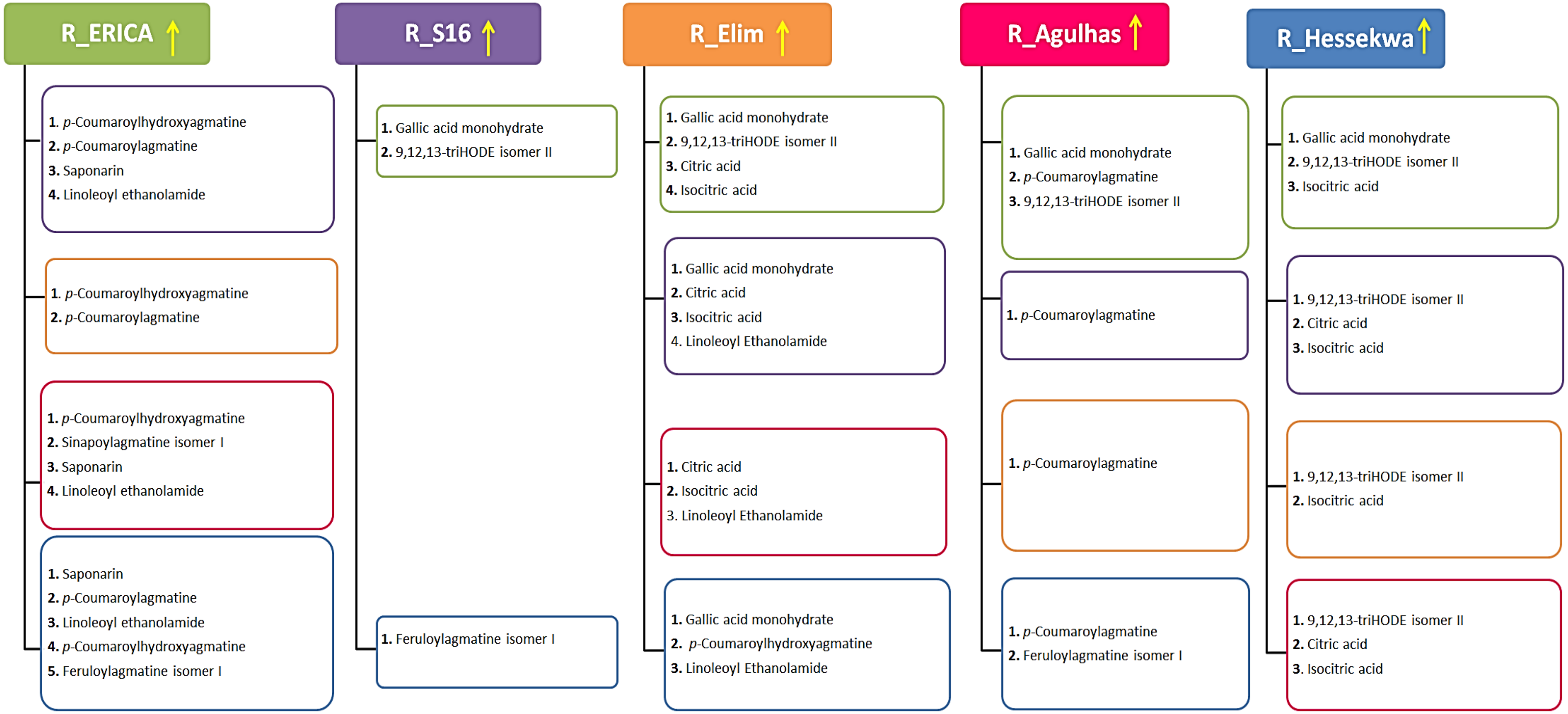

Figure S4. Discriminant metabolites selected on OPLS-DA S-plots generated from the comparison of extracts from roots of five cultivars with each other. The arrow indicates the positive correlation of the four groups of metabolites with the corresponding cultivars, e.g., in the first the column, all metabolites in the dark purple rectangle (corresponding to 'S16') are positively correlated with'Erica' and negatively correlated with 'S16'.

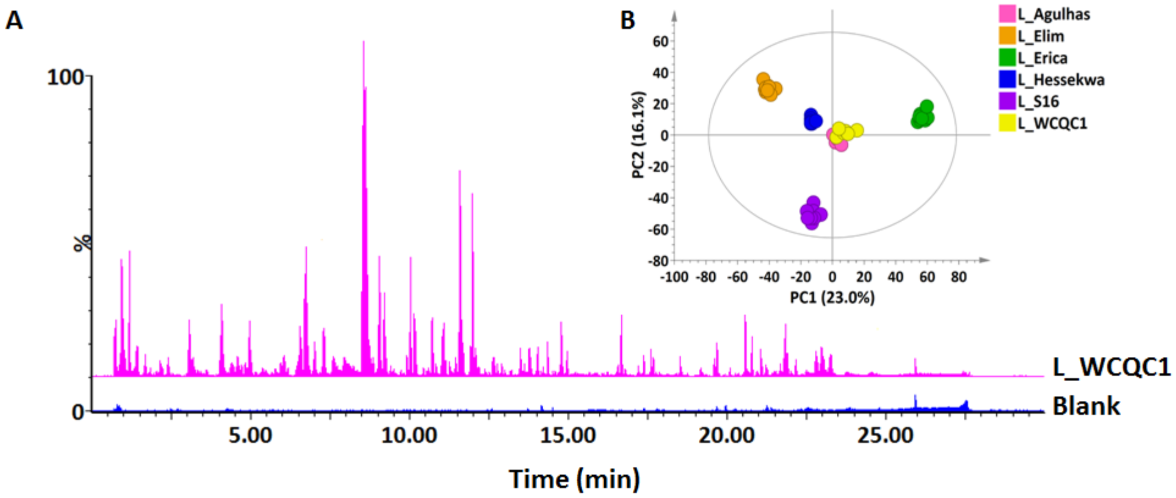

Figure S5. (A) Ultra-high performance liquid chromatography - mass spectrometry (UHPLC-MS) base peak intensity (BPI) chromatograms in negative electrospray ionisation (ESI) mode of blank and Western Cape quality control (WCQC1) samples. **(B)** A scores plot (PC1 *vs.* PC2) of a 4-component (PCA) model (*Pareto* scaled) explaining 57.6% variation and predicting 49.0% variation in leaves. The QC samples are clustering close to each other, indicating the stability of the LC-MS system and reproducibility of the analyses.
